# Some Public Aspects of the Wood-Alcohol Industry

**Published:** 1914-04-15

**Authors:** 


### SOME PUBLIC ASPECTS OF THE WOOD-ALCOHOL INDUSTRY.

Nearly a thousand cases of poisoning attributed to wood alcohol (mostly due to drinking it) have been reported in the literature since 1893, the date which marks the advent of methyl alcohol of a high grade of purity, like that sold under the trade names of "Columbian Spirits," "Colonial

Spirits," "Manhattan Spirits," "Pro Spirit," etc. The growing publicity of these distressing facts, accentuated by the agitation which has been widely fostered in print by THE JOURNAL among other publications, has directed attention to the danger from wood alcohol to such a degree as to arouse considerable uneasiness in the industries connected with its manufacture. We are informed from trustworthy sources[1] that the business involves the annual production and use of about ten million gallons of the substance with a capital investment in this country of about twelve million dollars in the industry, which employs over three thousand working people. It is interesting, therefore, to note the attitude taken in quarters in which business interests must inevitably permeate the purely humanitarian features that are also concerned. We are reminded that since man began to handle fire he has been utilizing dangerous substances to his own good purposes. It is, of course, true that many of the most useful substances are dangerous and poisonous. Deadly cyanids are used for extracting gold; poisonous strychnin is employed as a heart stimulant; toxic phenol finds wide application as a disinfectant, and corrosive acids are used in multitudinous operations.

Despite this indisputable statement, we cannot overlook the equally cogent fact that there are in all the cases mentioned inherent factors which of themselves limit the probability of harmful results in the uses of these poisons. Either there is an adequate understanding of the great dangers involved, or the applications are as a rule in the hands of experts who may be trusted to prevent untoward results. It is contended that methyl alcohol is used extensively as a valuable solvent and in the manufacture of many important materials, and that its legitimate use should not be prohibited. We may agree with these contentions and still raise the question whether or not in the case of the particular poison under discussion, methyl or wood spirits, the existing

1. These statistics are given on the authority of an editorial in the Journal of Industrial Engineering and Chemistry, 1913, v, 712.

legislation is adequate to prevent those disasters which experience has already demonstrated to occur.

It has been noted that wood alcohol presents a unique case for legislation, not only because of its general resemblance to ethyl alcohol, but especially on account of the word "alcohol," which has a definite meaning to the chemist, but is rather associated in the lay mind with "drink." The dangers, however, are by no means limited to the possibility of introducing methyl alcohol in foods or drinks. When, in 1906, the general agitation for a tax-free, denatured ethyl alcohol brought about hearings before the committees of Congress, the injurious action of wood alcohol on the general health and the eyesight of working people handling it in the industries was strongly emphasized by manufacturers employing it, workmen and experts. In the light of this it is interesting to learn the conclusions an recommendations of an unbaised report primarily arrived at from the point of view of the chemist and the industries rather than the alleged one-sided position of the hygienist. This is now available in the pamphlet on wood alcohol prepared for the New York State Factory Investigating Commission by Charles Baskerville, chairman of the Committee on Occupational Diseases of the American Chemical Society.[2] The report does not attempt to pass final judgment on the once debated question which has already been discussed in THE JOURNAL,[3] namely, whether the morbid action of wood alcohol is due to the concomitant impurities or not. Economic reasons prohibit the extended use of chemically pure methyl alcohol in the arts and manufacturing industries, although the purest material is now used in certain products. The abuses of methyl alcohol prior to the more recent activity in the direction of pure foods and drugs must be admitted,

---

2. Baskerville, Charles: Wood Alcohol: A Report on the Chemistry, Technology and Pharmacology of and the Legislation Pertaining to Methyl Alcohol. This can be secured by writing to the Commissioner of Labor, Albany, N. Y, An abstract appears in the Journal of Industrial Engineering and Chemistry, 1913, v, 768.

3. Methyl Alcohol as a Poison, editorial, THE JOURNAL A. M. A., Nov. 30, 1912, p. 1974.

and likewise the undoubted toxicity of the methyl alcohol of commerce.

There are at present sixty-three manufacturers of wood alcohol in the United States. In their plants the workmen are liable to come into contact with the vapor—one of the modes of intoxication—only in neutralizing the acetic liquor with lime and in filling the shipper containers. Baskerville maintains that general requirements for ample ventilation should meet these difficulties which, in fact, do not now exist in the works inspected in New York State. In the industries in which wood alcohol is employed it often serves as a solvent. Here it can exert deleterious action if the workmen (1) inhale the vapor; (2) dip their hands and arms into the liquor, or (3) drink it. Ample ventilation will avert the first danger; impervious gloves will prevent the second; only education can ward off the third. The precautionary measures are summarized in the New York report in the following recommendation as to laws which should be enacted if not already on the statute books:

1. To prohibit the presence of wood alcohol in any form of material intended for internal use.

2. To prohibit the presence of wood alcohol in preparations intended for external use on the human body.

3. To require ample ventilation in works in which wood alcohol is made or used in manufacturing products wherein the wood alcohol remains as such; the same law should apply where the products containing wood alcohol are used up, as for example, in varnishing vats in breweries.

4. To require containers in which wood alcohol is marketed to bear suitable display labels of warning.

After all is said, the fact remains that the manufacture and sale of Columbian Spirits and other forms of so-called "deodorized" methyl alcohol is a menace to the health of the people and ought to be suppressed. There is no valid argument for permitting its manufacture so long as an effective and cheap substitute exists. *Editorial in February 14, 1914, Journal A. M. A.*

